# Responses of Grassland Production to Single and Multiple Global Environmental Changes

**DOI:** 10.1371/journal.pbio.0030319

**Published:** 2005-08-09

**Authors:** Jeffrey S Dukes, Nona R Chiariello, Elsa E Cleland, Lisa A Moore, M. Rebecca Shaw, Susan Thayer, Todd Tobeck, Harold A Mooney, Christopher B Field

**Affiliations:** **1**Department of Global Ecology, Carnegie Institution of Washington, Stanford, California, United States of America,; **2**Department of Biological Sciences, Stanford University, Stanford, California, United States of America,; **3**Department of Plant Biology, Carnegie Institution of Washington, Stanford, California, United States of America; Ecole Normale SuperieureFrance

## Abstract

In this century, increasing concentrations of carbon dioxide (CO_2_) and other greenhouse gases in the Earth's atmosphere are expected to cause warmer surface temperatures and changes in precipitation patterns. At the same time, reactive nitrogen is entering natural systems at unprecedented rates. These global environmental changes have consequences for the functioning of natural ecosystems, and responses of these systems may feed back to affect climate and atmospheric composition. Here, we report plant growth responses of an ecosystem exposed to factorial combinations of four expected global environmental changes. We exposed California grassland to elevated CO_2_, temperature, precipitation, and nitrogen deposition for five years. Root and shoot production did not respond to elevated CO_2_ or modest warming. Supplemental precipitation led to increases in shoot production and offsetting decreases in root production. Supplemental nitrate deposition increased total production by an average of 26%, primarily by stimulating shoot growth. Interactions among the main treatments were rare. Together, these results suggest that production in this grassland will respond minimally to changes in CO_2_ and winter precipitation, and to small amounts of warming. Increased nitrate deposition would have stronger effects on the grassland. Aside from this nitrate response, expectations that a changing atmosphere and climate would promote carbon storage by increasing plant growth appear unlikely to be realized in this system.

## Abstract 2

The first five years of the Jasper Ridge Global Change Experiment show that production responses of the grassland to changes in climate and CO_2_ concentration are unlikely to lead to increased productivity on their own.

## Introduction

Since the start of the Industrial Revolution, human activities have changed the composition of the atmosphere at an accelerating rate, with increasingly recognized consequences for Earth's climate and biogeochemical cycles [1–3]. Ecosystem responses to these changes may further affect climate and biogeochemical cycling [3,4], and alter the character of ecosystem services provided to society [5]. During the past two decades, researchers have studied ecosystem responses to changes in climate, nitrogen (N) deposition, and atmospheric carbon dioxide (CO_2_) [6]. In some natural systems, responses of plant growth and resource use to one of these global changes have been extensively quantified. However, few studies have examined responses of ecosystems to the simultaneous and interacting global changes likely to be seen later this century. Even fewer studies have observed these responses over many years.

Production responses to single environmental changes vary widely among systems, and by year.

First, doubled atmospheric CO_2_ increased aboveground biomass production by an average of 14% across nine herbaceous systems [6]. However, CO_2_ enrichment suppressed production in some systems, while increasing it in others by as much as 85%. Some grasslands responded more positively in dry years than wet years [7–9], possibly because plants narrow their stomatal openings under elevated CO_2_, which leads to water savings.

Second, observed patterns of plant growth across natural gradients of precipitation and across years within locations suggest that increases in precipitation have the most positive effect on plant growth in systems with the lowest annual inputs [10]. Where precipitation exceeds about 3,000 mm per year, additional precipitation may suppress growth [11].

Third, warming increases aboveground biomass production in many systems, with the strongest effects in colder climates. Across 20 experimental warming sites in tundra, grassland, and forest, increases in aboveground productivity averaged 19% [12]. Across natural systems, production tends to increase with increasing mean annual temperature [11]. Within some productive systems, aboveground growth is correlated with maximum growing season temperature [10].

Fourth, responses to N additions are generally positive across temperate, boreal, and arctic systems [13,14].

While all terrestrial systems are experiencing a fairly uniform increase in CO_2_, the character of other global changes varies from one region to the next. Thus, the mix of global changes impacting a given region will depend on both space and time. Understanding the responses of ecosystems to potentially interacting global changes is critical to predicting ecosystem feedbacks to climate and biogeochemical cycles. In particular, the response of carbon (C) storage in ecosystems is dependent on (and proportionally related to) two ecosystem processes: C inputs from primary production, and the residence time of C in the system [15].

Several previous studies have examined interactions between N availability and other global change factors [16–18], and some have examined interactions between CO_2_ and changes in water availability [8,9], climate [19–21], or loss of biodiversity [22,23]. However, we are still developing a conceptual framework to describe the conditions under which a given interaction is most important. For instance, mineral element availability may progressively limit positive CO_2_ responses in some systems, but other systems are unlikely to develop such an interaction [24]. Similarly, where elevated CO_2_ leads to important soil moisture savings [25], increases in precipitation might negate any CO_2_ effect. Temperature and CO_2_ responses are frequently assumed to be additive, although few ecosystem-scale experiments exist [26]. No previous studies, to our knowledge, have simultaneously tested responses to enhanced CO_2_, warming, increased precipitation, and increased N deposition.

Since 1998, the Jasper Ridge Global Change Experiment (JRGCE) has exposed a moderately fertile grassland to atmospheric and climate conditions expected later this century, and to enhanced nitrate deposition. Because small-statured, annual species dominate California grasslands, this ecosystem is well suited for the study of responses to global changes. Thousands of individual plants can be examined within a small area, and changes in the chemistry of plants and plant litter quickly reach the soil as the plants die. Additionally, the plants complete one generation each year, so competition and selection can “tune” the performance of the grassland to new environmental conditions more quickly than would occur in systems with longer-lived species. While systems dominated by larger, longer-lived organisms might adjust to a step change in CO_2_ or N deposition over a span of decades, annual grassland can be expected to reach a steady, “representative” response more quickly.

With a wide range of treatments and treatment combinations, the JRGCE provides a foundation for characterizing how ecosystems may perform in the future in a range of possible scenarios. Of particular interest is determining whether ecosystem responses to individual factors are additive. How reliably can we predict ecosystem responses to many concurrent environmental changes based on responses to individual changes? Previously, Shaw et al. [27] focused on CO_2_ responses in this grassland and found an unexpected result: elevated CO_2_ suppressed positive production responses to other global changes during the third year of the JRGCE. Here, we present a comprehensive description of the responses of grassland production to all four global changes over the first 5 y of experimental treatments, and discuss these responses in the context of natural, as well as experimental, climate variation. With this expanded dataset, we are able to put the results from Shaw et al. [27] in a larger context, and determine whether there have been consistent changes in grassland net primary production (NPP) that could directly affect the amount of C stored in this ecosystem.

## Results

### Production Responses to Global Changes

**Figure 1:**
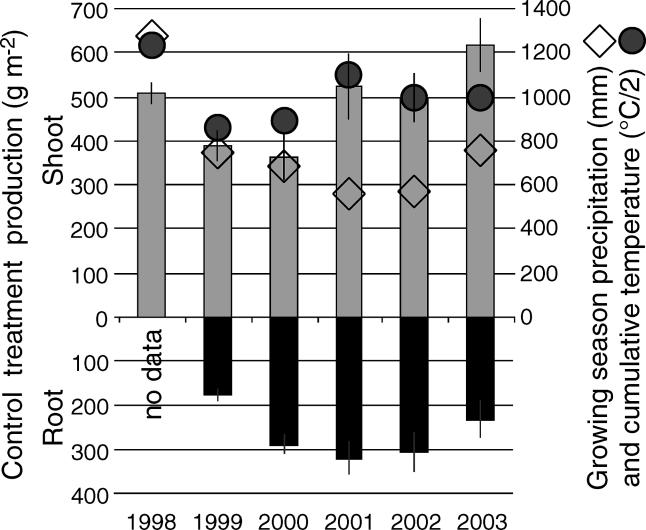
Biomass Production, Cumulative Precipitation, and Cumulative Temperature from 1998 (the Year before Treatments Began) to 2003 Bars for shoot biomass (grey) and root biomass (black) represent mean values (±SE, *n* = 8) for quadrants in the “infrastructure control” treatment, which experienced ambient conditions (see Materials and Methods). Cumulative precipitation includes the germinating rain event (defined here as the event that brought cumulative rainfall after October 1 above 12.5 mm) and subsequent precipitation until the last harvest of the year. Cumulative temperature is the sum of average temperatures (in °C) over all days from germination until the final harvest. Shoot biomass (g m^−2^) was not related to growing season precipitation (mm; linear regression: *p* = 0.87) or cumulative temperature (°C; *p* = 0.27), but there was a weak relationship between shoot growth and fall precipitation (linear regression: *p* = 0.06, slope = 0.646, *r* ^2^ = 0.627).

**Figure 2:**
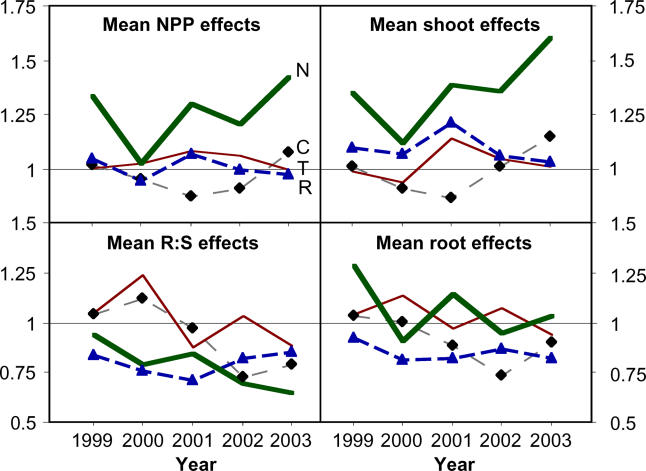
Proportional Responses of NPP (Measured as Root + Shoot Biomass), Shoot Biomass, Root Biomass, and Root-to-Shoot Ratio to the Four Global Change Treatments Each line represents the response over time to a single global change factor, and each data point represents the sum of eight elevated treatment averages divided by the sum of eight ambient treatment averages. Elevated CO_2_, C (gray dashed line and filled diamonds); increased temperature, T (thin red solid line); increased rainfall, R (thick blue dashed line and triangles); nitrate deposition, N (thick solid green line).

**Figure 3:**
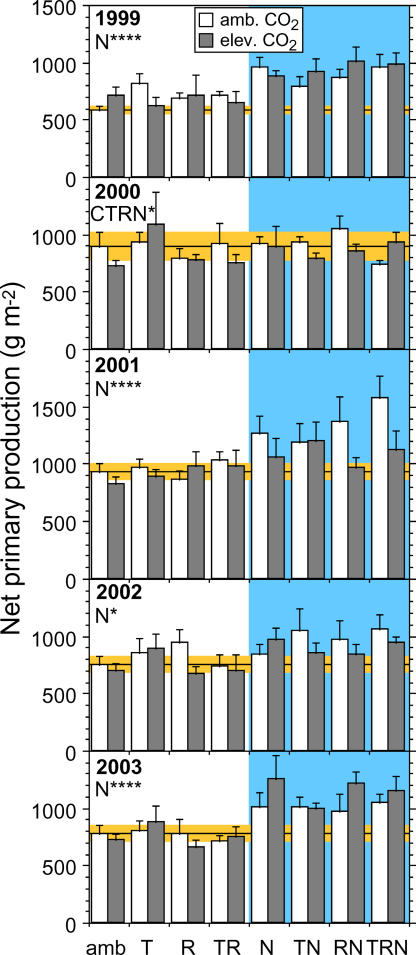
NPP (Measured as Root + Shoot Biomass) in Individual and Combined Global Change Treatments Note that in the similar Figure 2 of Shaw et al. [27], the reference treatments were not the plots with all global change factors at ambient levels, but the average of all of the plots with the global-change factors identified under each bar at ambient levels. Note also that while Shaw et al. eliminated two blocks heavily invaded by non-native perennials in 2000–2001, the current analysis uses data from all of the blocks from every year. Paired bars depict mean values under ambient (open) and elevated CO_2_ (grey). A line representing mean biomass of the ambient treatment is drawn across each panel to facilitate comparisons. Shaded yellow areas similarly mark zones within one standard error of the ambient treatment. Panels are shaded blue behind treatments receiving N to highlight plant responses to this treatment. Letters in each panel identify treatment effects (α < 0.05; C denotes elevated CO_2_) from mixed model analyses of NPP data (*, *p* < 0.05; **, *p* < 0.01; ***, *p* < 0.001; ****, *p* < 0.0001). Interactions are presented as multiple letters. Ambient, amb; increased temperature, T; increased rainfall, R; nitrate deposition, N. Error bars denote one standard error.

Mean production (NPP) of the control treatment varied from 577 to 933 g m^−2^ across the 1999–2003 growing seasons (see related data in Figure 1). The four main treatments differed in their average effects on NPP (Figures 2 and 3). Only nitrate deposition consistently affected NPP, causing increases of 21%–42% in all years but 2000. Shoots generally responded more positively to N addition than roots did, leading to decreases in root-to-shoot ratios (Figure 2; Tables S1 and S2). Increased precipitation had little effect on NPP, as negative root responses largely counteracted positive shoot responses. Neither warming nor elevated CO_2_ significantly affected shoot, root, or total production in any year. Increased precipitation and nitrate deposition frequently decreased root-to-shoot ratios, while heat and CO_2_ did not affect allocation (Figure 2; Tables S1 and S2).

**Figure 4:**
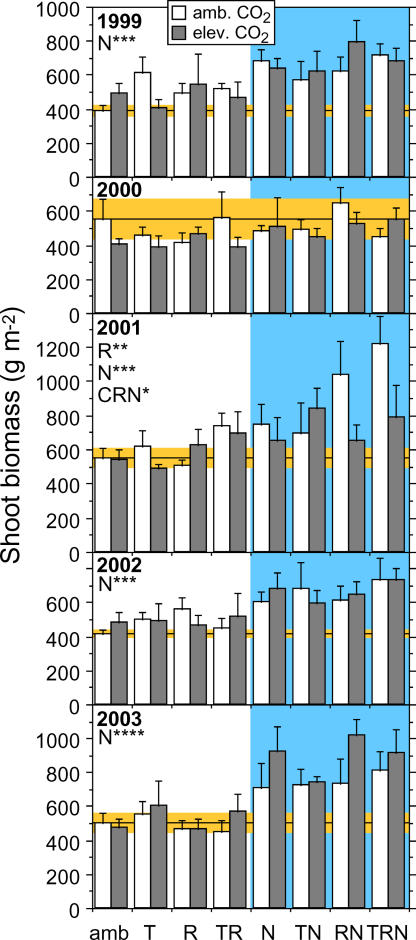
Mean Shoot Biomass in Individual and Combined Global Change Treatments Error bars denote one standard error. Shading, labels, and statistics as in Figure 3.

**Figure 5:**
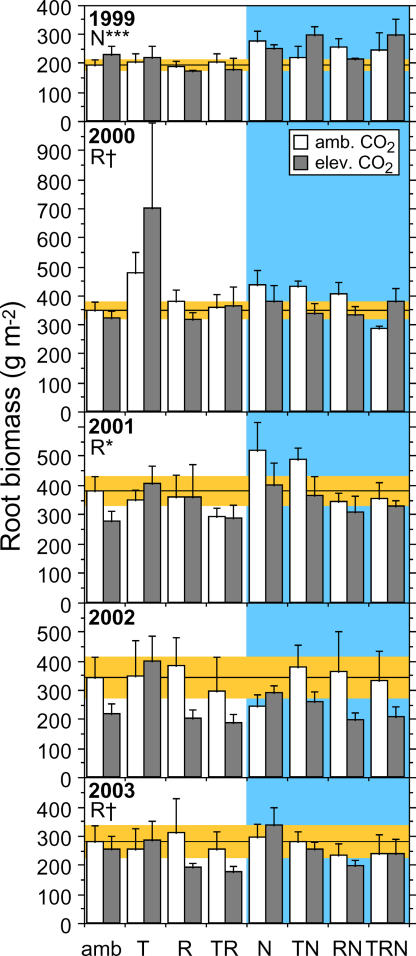
Mean Root Biomass in Individual and Combined Global Change Treatments Error bars denote one standard error. Shading, labels, and statistics as in Figure 3, with one addition (†, 0.10 > *p* > 0.05).

Nitrate strongly increased NPP in all years but 2000, when a four-way interaction was significant (Figure 3; Table S3). Aboveground biomass responses drove the NPP results, as nitrate increased shoot production in all years but 2000 (Figure 4; Table S4). In 2001, nitrate also affected shoot responses to rainfall and CO_2_; precipitation responses were more positive under increased N, but only in ambient CO_2_ levels. In this year only, elevated CO_2_ suppressed the shoot response to combined N and precipitation (Figure 4). Roots responded positively to nitrate deposition in 1999, but not in subsequent years. In 2000, 2001, and 2003, increased rainfall suppressed root production (Figure 5; Table S5).

**Figure 6:**
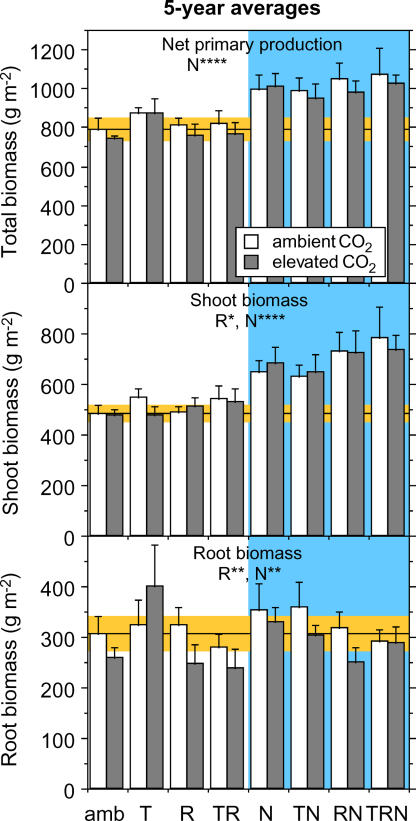
Mean Total Biomass, Shoot Biomass, and Root Biomass in Individual and Combined Global Change Treatments during the 1999–2003 Period Shading and labels as in Figure 3. Letters identify treatment effects (α < 0.05) from a repeated measures mixed model analysis (*, *p* < 0.05; **, *p* < 0.01; ****, *p* < 0.0001). Error bars denote one standard error.

Across all 5 y of the experiment, nitrate deposition strongly increased shoot biomass and NPP, and slightly increased root biomass (repeated measures analysis; Figure 6). The only other treatment to affect biomass production across years was precipitation, which increased shoot growth but suppressed root growth, leading to no effect on NPP (Figure 6).

### How Are Responses to Global Changes Affected by the Background Climate?

In most cases, grassland responses to the global change treatments did not depend on climatic factors as measured by regressions against accumulated degree-days or total precipitation (*p* > 0.05). The exception was the response of shoot growth to temperature, which increased in warmer years (*p* < 0.05, slope = 0.001, *r* ^2^ = 0.10). While the total precipitation and degree-day sums facilitate simple comparisons of responses across years and climate treatments, these metrics do not necessarily capture the critical aspects of climate during the growing season. The sensitivity of grassland production to interannual variation in weather is further discussed below.

### Progressive Effects

The pattern from 5 y of treatments in the JRGCE hints at the possibility of progressive or cumulative effects but is rarely definitive. For NPP, aboveground production, and belowground production, no regression of treatment effect on time is significant. Across all of the single-factor treatments and treatment combinations, treatment effects appeared to progressively decrease root production (*p* = 0.09). The strongest evidence for progressive effects comes from changes in allocation patterns. The effect of nitrate on root-to-shoot ratios became increasingly negative over time (*p* = 0.02), and the response of root-to-shoot ratios to elevated CO_2_ shifted from positive to negative (*p* = 0.07).

## Discussion

Over the first 5 y of the JRGCE, production responded minimally to elevated temperature and CO_2_, positively to increased nitrate deposition, and in a context-dependent manner to supplemental precipitation. The response of production to elevated CO_2_ is negative in some but not all treatments and years [27]. As a consequence, NPP did not respond to elevated CO_2_ over the 5-y period (means: ambient CO_2_, 925 g m^−2^; elevated CO_2_, 887 g m^−2^; *p* = 0.62). The lack of response to CO_2_ enrichment indicates that this and similar grasslands are unlikely to experience strong C sinks from CO_2_ fertilization over the next century. Neither is the response to any future precipitation increase likely to lead to increased C storage. Future increases in nitrate deposition could increase NPP and perhaps C storage, while the experimental warming was probably too slight to interpret as an analog for the end of the century.

### Responses to Global Environmental Changes

Except in groups of treatments [27], production did not respond significantly to elevated CO_2_ (*p* > 0.1). This suggests that our system exhibits one of the lower grassland NPP responses [9], but is consistent with results from an earlier open-top chamber study of CO_2_ effects on a neighboring patch of California grassland. In that experiment, production also was not affected by CO_2_ enrichment [28]. In the same study, NPP of an adjacent patch of serpentine grassland increased in response to elevated CO_2_ (averages: +12% and +29% in 2 y), largely as a result of increased growth by a few summer-active species that take advantage of wetter soils in late spring. In the JRGCE, such late-flowering forbs were rare. They were absent from all harvested areas in 1999 and constituted 0.37% of the aboveground biomass harvest in 2003. Our peak biomass harvests took place before these forbs reached full size, but the species were so rare that their responses could have impacted overall NPP only if they were massively sensitive to the observed water savings from elevated CO_2_ [29].

Warming did not affect grassland production, but it did affect the phenology of many species ([30]; Chiariello et al., unpublished data). Zavaleta et al. [29] found that warming led to earlier senescence of many of the dominant species, leaving additional water in soils over the summer. In a setting with responsive plant species, this water savings could combine with that from elevated CO_2_ to increase the production and/or establishment of late-season annuals, shrubs, and trees [31].

Precipitation affected plant growth more strongly than warming or CO_2_, with positive effects on shoots and negative effects on roots across years (Figure 6), although analyses of individual years indicated that these effects were not always significant (see Figures 2 and 5). Averaged across treatments, these counteracting shoot and root effects led to no effect on NPP (see Figures 3 and 6). Why did supplemental precipitation decrease root growth? It is possible that allocation to roots decreased as soil resources became more available. In this case, root growth could have been downregulated by increases in water availability or availability of nutrients that are mobile in water. A leading candidate for such a nutrient would be nitrate, which consistently decreased root-to-shoot ratio, with significant effects in two of the 5 y. It is also possible that root growth is affected by small changes in water availability, or that soils in the precipitation treatment occasionally became waterlogged, suppressing root respiration.

Of the four global changes, nitrate deposition had the most consistent, positive effects on plant production. Nitrate increased shoot growth more consistently and by a greater amount than root growth, leading to lower root-to-shoot ratios. Several previous studies have found similarly positive responses of California grasslands to various forms of N (e.g., [32–35]).

### Explaining the CO_2_ Response

The biomass responses presented here include the results from the 2000–2001 growing season discussed by Shaw et al. [27]. The analysis by Shaw et al. primarily focused on CO_2_ responses, demonstrating that elevated CO_2_ partially suppressed NPP increases in response to warming, extra precipitation, and nitrate deposition. Across the 5-y dataset, as in the analysis of Shaw et al., there was not a significant, experiment-wide CO_2_ effect over the entire dataset or in any individual year.

Why doesn't elevated CO_2_ increase production in this grassland? Several lines of evidence suggest phosphorus (P) limitation could play a role. Both N deposition and elevated CO_2_ decrease plant P concentration, and, at least in some treatment combinations, elevated CO_2_ reduces total plant P uptake [30]. In some years, elevated CO_2_ appears to favor P uptake by microbes over plants [30]. A comparison of grassland production responses after a summer wildfire at the JRGCE also supports the P limitation hypothesis (H. Henry et al., unpublished data). In this study, elevated CO_2_ suppressed production in unburned grassland, but not in burned areas. Plant growth also responded more strongly to N deposition in burned areas. Ratios of N to P in shoots of the dominant annual grasses were lower in the burned area, suggesting that the fire may have made available more P in ash deposits. The burn alone did not increase plant growth, indicating P limitation may be triggered by elevated CO_2_ or N deposition. Henry and colleagues cannot definitively separate effects of P availability from changes in microclimate in the burned area, but further studies on the role of P in this grassland are under way. Ongoing research in the JRGCE is also exploring how changes in herbivory, phenology, allocation, and other factors may prevent the grassland from responding positively to CO_2_.

### The Role of Weather

Rangeland scientists have developed many equations to predict shoot growth (forage yields) in annual grasslands based on weather variables [36–40]. In general, measures of heat (as degree-days) and/or precipitation predict annual shoot growth with reasonable accuracy [39,40]. Warmer years and wetter fall and spring seasons usually lead to greater shoot growth. Why, then, did increased temperatures and precipitation not consistently increase shoot growth in this experiment? Most of the precipitation additions in this experiment were associated with rain events, and most of these events occurred in the colder months of winter. In contrast to precipitation in the fall and spring, winter rainfall is not significantly related to shoot production in California grasslands ([36]; J. Dukes, unpublished data). Supplemental precipitation could have its greatest effect by advancing the start of the growing season, eliminating occasional mid-season droughts, or delaying the end of the growing season in years with dry spring months. Our precipitation treatments never advanced the start of growing seasons, but occasionally reduced drought severity. The most positive effect of precipitation was in 2001 (see Figure 2), when a dry period occurred at the end of the growing season and the added precipitation extended the growing period in addition to eliminating the drought.

Despite predictions that CO_2_ responses would be most positive in drier years [7], this was not the pattern in the JRGCE. Across the range of annual precipitation that the ambient and watered treatments experienced from 1999 through 2003, there was no trend in CO_2_ response.

### Progressive Effects

Responses of NPP to global changes could be progressive for a number of reasons. Changes in community structure could lead to increases in the abundance of unusually responsive (or unresponsive) species. Continuing additions may directly affect the availability of a potentially persistent resource (e.g., nitrate). Or there might be feedbacks through the quantity or quality of soil organic matter [24]. The strengthening effect of N deposition on root-to-shoot allocation patterns could result from a progressive increase in N availability as a consequence of an increasing ecosystem stock. It may also reflect stimulated N mineralization resulting from increased rates of decomposition [41].

In the JRGCE, the evidence for progressive effects is limited in the results to date. Tests to quantify the magnitude, direction, and persistence of progressive effects are a central goal of continuing studies.

### Other Considerations

The change in the harvest strategy from one to two harvests between 2000 and 2001 may account for subtle differences between the responses in the first two and the last three years of the dataset. In some cases, responses to treatments were stronger at the second harvest than in the first. High variability in harvested root biomass complicates the task of quantifying treatment effects on root production. Frequently, variation within a treatment was extreme. For instance, in 2003 the control treatment averaged 278 g m^−2^ root biomass, with a range of 122–552 g m^−2^.

Our biomass-based root production results underestimated actual production for two reasons. First, annual root production exceeds peak live biomass by approximately 50% in this grassland [42]. Second, we used root data from soil cores taken to 15 cm, a depth that captures 80%–90% of total root biomass (L. Moore, unpublished data). To assess the impacts of these simplifications, we recalculated our data using correction factors. First, we estimated root biomass to 30 cm. For three of the five years, we had measured root biomass from soil cores to 30 cm depth. In the other 2 y, we estimated peak root biomass to 30 cm based on the ratio of roots to 15 cm and 30 cm in the years for which we had measurements. Second, we multiplied the biomass to 30 cm by 1.54, to account for turnover [42]. These corrections had little effect on patterns of NPP responses to the treatments, and altered the significance of the results in only two cases (the temperature × nitrate interaction became significant [*p* = 0.047] in 2000, and the nitrate effect became only marginally significant [*p* = 0.086] in 2002).

### Implications

What are the implications of these changes in grassland production for C storage and other ecosystem services? Production increases are a likely requirement for greater C storage, although the relationship between NPP and storage depends on several other factors that affect C in soils. Shoot biomass provides forage for livestock and wildlife, and influences fire behavior in wildlands and urban/wildland interfaces [43]. Results from the first 5 y of the JRGCE suggest that the rising atmospheric CO_2_ concentration will have small and year-dependent effects on production. Across all treatments and years, total grassland production did not respond to CO_2_ enrichment. This overall lack of sensitivity suggests that, over the long term, effects of CO_2_ on ecosystem services are more likely to occur through secondary responses such as changes in tissue chemistry, soil moisture, and species composition than directly through changes in production. In individual years, however, the CO_2_ response may alter production in a meaningful way [27]. Our warming treatment was quite modest, and the lack of a production response to this small temperature change does not suggest that the system will be insensitive to the greater warming likely to occur by 2100. Additionally, effects of our warming treatment on the phenology of grassland species could have important consequences for forage availability and the duration of the wildfire season ([30]; Chiariello et al., unpublished data). Responses of the fungal community to warming [44] may also be important for C storage. Increases in growing season precipitation led to small changes in shoot production, but acted to strongly decrease root production. As with the other factors, whether precipitation leads to greater C storage under any scenario will depend on the responses of other processes, such as microbial respiration. Of the four global change treatments, nitrate deposition had the most consistent and most positive effects on shoot biomass and total biomass production. These production increases, which averaged 37% and 26%, respectively, have obvious potential to meaningfully alter ecosystem services.

The first 5 y of the JRGCE show that production responses of the grassland to changes in climate and CO_2_ concentration are unlikely to lead to increased productivity on their own. While interactions among changes in climate and CO_2_ may influence biomass production in specific years, the consequences of these interactions appear limited when averaged over longer time scales.

The JRGCE is one of the most comprehensive global-change experiments to date. It is one of relatively few ecosystem scale experiments to use naturally occurring—as opposed to artificially constructed—ecosystems. We see no reason to think that the kinds of responses observed in the JRGCE are not quite general, at least for natural communities in temperate climates on soils of moderate nutrient availability. Comparing the single-factor responses in the JRGCE with single-factor responses in other ecosystems should provide an efficient approach for assessing the generality of the JRGCE responses to simulated global changes.

## Materials and Methods

### Study site and system

The JRGCE is located in Jasper Ridge Biological Preserve, near Woodside, California, United States (37°24′N, 122°14′W, 120 m elevation). This region experiences a Mediterranean climate, with cool, wet winters and warm, dry summers. The experiment was conducted in 36 plots dispersed across ∼0.75 ha of natural grassland. Each plot was circular, 2 m in diameter, and divided into four equal-sized quadrants. The dominant species in this location are typically annual grasses *(Bromus hordeaceus, Avena barbata, A. fatua)* and annual forbs *(Geranium dissectum, Erodium botrys).* Perennial grasses and forbs are common but rarely dominant. A few of the ∼35 herbaceous species present in the study area increased in dominance over the course of the experiment, most prominently the perennial grass *Danthonia californica* and the biennial forb *Crepis vesicaria* ssp. *taraxifolia*.

Plots were established in the summer of 1997, with the 1997–1998 growing season used as a pretreatment year. The exceptional rainfall and warmth of the 1997–1998 El Niño (see Figure 1), however, made this year somewhat unusual. From 1974–2003, the site received an annual average of 655 mm precipitation (as measured by a weather station located within 1 km of the experimental area).

### Global change treatments

In a complete factorial design, we exposed the grassland to ambient and elevated levels of four factors: atmospheric CO_2_, temperature, precipitation, and nitrate deposition. Experimental treatments were imposed during every growing season (roughly November to June) starting in fall 1998. We added heat and CO_2_ at the whole-plot level. Free-air CO_2_ enrichment (FACE; [45]) was used to elevate atmospheric CO_2_ to ∼680 μmol mol^−1^. Single 250-W infrared heaters, suspended 1 m over the center of each warming treatment plot, operated continuously during the growing season from 1998–2002. In the 2002–2003 growing season, the central infrared heater was replaced by four 60-W heaters, each centered over one quadrant. In both cases, the heaters elevated canopy/air temperatures by approximately 1 °C. Unheated plots were equipped with “dummy” heaters to reproduce any shading or other effects of the heaters. Precipitation and nitrate deposition were supplemented factorially at the quadrant level. Quadrants in the increased precipitation treatment received 150% of the annual rainfall, with precipitation supplemented via drip tubing (1998–1999) or overhead sprinklers (1999–2003) shortly after each rain event. In addition, two rain events (20 mm each) were applied at the end of each growing season to extend the rainy season by 3 wk. Nitrate was applied twice per year as Ca(NO_3_)_2_. Early in the growing season (November), 2 g N m^−2^ was added in solution to mimic the flush of accumulated dry deposition N that enters the system with the first rains. Later in the season (January–February), 5g N m^−2^ was added as slow-release pellets (Nutricote 12–0–0, Agrivert, Riverside, California, United States). Fiberglass barriers (0.5 m deep) separated soil in neighboring quadrants, and kept soil in the plot separate from soil in the surrounding grassland. Above the surface, vertical sections of netting discouraged plants, seeds, and plant litter from crossing quadrant boundaries. This netting had a minimal effect on light, reducing the intensity of incoming photosynthetically active radiation by less than 5% (Sunfleck Ceptometer, Decagon Devices, Pullman, Washington, United States).

A separate set of four 2-m diameter “infrastructure control” plots were demarcated in the field but were not equipped with soil partitions, netting, heaters, or CO_2_ and water distribution tubes. These plots experienced ambient conditions throughout the experiment. Plant production was measured as described below in two quadrants of each infrastructure control plot.

### Treatments vs. predicted global changes

The treatments in this experiment were selected not only to simulate conditions predicted to occur in the region within the next century, but also to allow a more comprehensive understanding of mechanisms driving the grassland responses, their relevance to other ecosystems, and their likely limits. Modulation of CO_2_ sensitivity by availability of other resources is a dominant but unresolved theme in global change research [6]. Variation in resource availability is a central motif across a broad range of global change studies, and along with climate and species composition, may prove useful in generalizing responses across ecosystems. Consistent patterns of response in relation to resource availability could lay the foundation for extending results to other locations, time periods, or management regimes.

To place our treatment levels in the context of recent predictions, atmospheric CO_2_ concentrations could reach 680 ppm as early as 2070 [2]. In California, climate is expected to warm by 2.3–5.8 °C within this century [46]. Our warming treatment is therefore quite conservative, and will likely be exceeded by mid-century or earlier [46]. Our precipitation treatment simulates slightly smaller increases than predicted by the Hadley Climate Model, version 2, for 2080–2099 [47,48], although projections with newer models tend to be somewhat drier (cf. [46]). Our N deposition treatment was designed primarily to help determine whether N availability limits grassland responses to other global changes. Jasper Ridge currently receives approximately 0.5 g N m^−2^ yr^−1^ [49], but other areas of the world receive rates of deposition approaching or exceeding our treatment [50]. Taken together, the treatments used here provide a broad range of combinations of conditions that may occur both locally and in diverse sites, within this century.

### Production measurements

We estimated NPP by summing measurements of live and senesced shoot and live root biomass within each quadrant. Because annual plants dominate the grassland, measurements made at the time of peak biomass (mid April to late May, depending on the treatment and year) provide reasonable estimates of aboveground and belowground production, though without including the transfer of C to mycorrhizae, root exudation, and root turnover during the growing season [42]. In 1998 (the pretreatment year), 1999, and 2000, we harvested aboveground biomass once. Beginning in 2001, we conducted two aboveground biomass harvests approximately 1 mo apart, to increase our chances of capturing the maximum value for the season in each quadrant. For years with two aboveground harvests, we used the maximum value from each quadrant to estimate NPP. During each aboveground harvest, we collected all aboveground plant matter in a 141 cm^2^ area, and separated the current year's production from that of previous years. Biomass from the first (or only) harvest was separated by species before weighing. Starting in 1999, root biomass was determined by separating live roots out of soil cores (15 cm depth) taken in the area of the first aboveground biomass harvest, shortly after the harvest. All biomass was oven-dried (70 °C) before weighing. We refer to data from each growing season by the year in which the harvests occurred.

### Statistical analysis

Treatment effects for our experimental design were analyzed with a full factorial, split-plot model using the PROC MIXED method of maximum likelihood estimation in SAS (SAS v.8, Cary, North Carolina, United States). Warming and elevated CO_2_ treatments were included as whole-plot effects, and precipitation and nitrate deposition treatments were included as split-plot effects. We tested for treatment effects with the restricted maximum likelihood method, using the containment method for determining degrees of freedom and using ordinary least squares starting values where necessary. For analyses of the full 5 y of NPP data, we used PROC MIXED to run a repeated measures version of the split-plot model, in which we used default starting values, unstructured covariance, and the Kenward-Roger technique for determining denominator degrees of freedom. Unless otherwise indicated, values were untransformed (in these cases, analyses with log-transformed data provided virtually identical results). Other statistical techniques are discussed with the results. We excluded data from 13 quadrants in the 1999 dataset, due to errors in the application of the nitrate treatment and inconsistencies in the output of one heater. In all other years, all 128 quadrants were analyzed.

To examine whether grassland responses to global change treatments were dependent on climatic factors, we regressed proportional responses of shoots, roots, and total biomass to the global change treatments against accumulated degree-days (°C) and growing season precipitation (mm). Proportional response values were calculated using treatment means. Means from treatments with elevated levels of a factor were divided by means of the corresponding treatments with ambient levels of that factor. For instance, when regressing the N response against precipitation, one proportional response value was the mean of the CTN (elevated CO_2_, temperature, and nitrogen) treatment divided by the mean of the CT (elevated CO_2_ and temperature) treatment. The rainfall and heating treatments caused four data points to fall on each of two values on the independent axis (precipitation or temperature) each year. For each regression, *n* = 40.

## Supporting Information

Table S1 Results (*p*-Values) from Mixed Model Analyses of Treatment Effects on Root-to-Shoot Ratio (ln-Transformed) Because these analyses used the containment method to determine denominator degrees of freedom (DDF), all treatments in a given year had the same number of DDF. Values for DDF from 1999–2003 were 23, 28, 28, 28, and 27, respectively. Elevated CO_2_, C; increased temperature, T; increased rainfall, R; nitrate deposition, N. Numerator degrees of freedom: 1 (all years).
(43 KB DOC).

Table S2 Results (*p*-Values) from Mixed Model Repeated Measures Analysis of Treatment Effects on Root-to-Shoot Ratio (ln-Transformed) Treatment labels as in Table S1.
(36 KB DOC).

Table S3 Results (*p*-Values) from Mixed Model Analyses of Treatment Effects on NPP Treatment labels as in Table S1. Numerator degrees of freedom: 1 (all years). Denominator degrees of freedom (1999–2003): 23, 28, 28, 28, 27, respectively.
(42 KB DOC).

Table S4 Results (*p*-Values) from Mixed Model Analyses of Treatment Effects on Aboveground Production Treatment labels as in Table S1. Numerator degrees of freedom: 1 (all years). Denominator degrees of freedom (1999–2003): 23, 28, 28, 28, 28, respectively.
(42 KB DOC).

Table S5 Results (*p*-Values) from Mixed Model Analyses of Treatment Effects on Belowground Production Treatment labels as in Table S1. Numerator degrees of freedom: 1 (all years). Denominator degrees of freedom (1999–2003): 23, 28, 28, 28, 27, respectively.
(43 KB DOC).

## Abbreviations

C: carbon
CO_2_: carbon dioxide
JRGCE: Jasper Ridge Global Change Experiment
N: nitrogen
NPP: net primary production
P: phosphorus
