# Supplementary material for: Responses of Grassland Production to Single and Multiple Global Environmental Changes
Source: PLoS Biol. 2005 Aug 9;3(10):e319. doi: 10.1371/journal.pbio.0030319 (PMC1182693; doi:10.1371/journal.pbio.0030319)
Supplement: Supplementary file 1 [file pbio.0030319.st001.doc]

Table S1. Results (*P*-values) from mixed model analyses of treatment effects on root : shoot ratio (ln-transformed)^1^

| Year | 1999 | 2000 | 2001 | 2002 | 2003 |
| --- | --- | --- | --- | --- | --- |
| Treatment |  |  |  |  |  |
| C | 0.5629 | 0.7594 | 0.9688 | 0.3041 | 0.3334 |
| T | 0.9626 | 0.3469 | 0.4353 | 0.9291 | 0.5053 |
| C*T | 0.2139 | 0.5674 | 0.5755 | 0.7181 | 0.7744 |
| R | *0.0949* | *0.0501* | **0.0010** | **0.0385** | *0.0656* |
| C*R | 0.3596 | 0.5601 | *0.0969* | 0.1837 | 0.2210 |
| T*R | 0.9233 | 0.4485 | 0.3246 | 0.1880 | 0.8149 |
| C*T*R | 0.9861 | 0.4680 | 0.4673 | 0.6338 | 0.8724 |
| N | 0.4567 | 0.1851 | 0.3222 | **0.0164** | **0.0095** |
| C*N | 0.5589 | 0.7625 | 0.8197 | 0.7395 | 0.7218 |
| T*N | 0.4544 | 0.2153 | 0.8708 | 0.8971 | 0.6203 |
| C*T*N | 0.6932 | 0.2421 | 0.1158 | 0.1520 | 0.6144 |
| R*N | 0.7051 | 0.5386 | 0.3553 | 0.8908 | *0.0923* |
| C*R*N | 0.9861 | 0.3165 | *0.0554* | 0.7859 | 0.5926 |
| T*R*N | 0.5485 | 0.1559 | 0.2579 | 0.8599 | 0.7350 |
| C*T*R*N | 0.3306 | 0.9049 | 0.2291 | 0.1792 | 0.2814 |

^1^Treatment labels: elevated CO_2_, C; increased temperature, T; increased rainfall, R; nitrate deposition, N. Numerator degrees of freedom: 1 (all years). Because these analyses used the containment method to determine denominator degrees of freedom (DDF), all treatments in a given year had the same number of DDF. Values for DDF from 1999-2003 were 23, 28, 28, 28, and 27, respectively.
