# Supplementary material for: Responses of Grassland Production to Single and Multiple Global Environmental Changes
Source: PLoS Biol. 2005 Aug 9;3(10):e319. doi: 10.1371/journal.pbio.0030319 (PMC1182693; doi:10.1371/journal.pbio.0030319)
Supplement: Supplementary file 2 [file pbio.0030319.st002.doc]

Table S2. Results (*P*-values) from mixed model, repeated measures analysis of treatment effects on root : shoot ratio (ln-transformed)^1^

| Year | Numerator DF | Denominator DF | P-value |
| --- | --- | --- | --- |
| Treatment |  |  |  |
| C | 1 | 25.7 | 0.7255 |
| T | 1 | 25.7 | 0.9710 |
| C*T | 1 | 25.7 | 0.3235 |
| R | 1 | 80.6 | **<0.0001** |
| C*R | 1 | 80.6 | 0.3546 |
| T*R | 1 | 80.6 | 0.2204 |
| C*T*R | 1 | 80.6 | 0.6052 |
| N | 1 | 81.2 | **0.0031** |
| C*N | 1 | 81.2 | 0.5195 |
| T*N | 1 | 81.2 | 0.9703 |
| C*T*N | 1 | 81.2 | 0.2294 |
| R*N | 1 | 80.6 | 0.2668 |
| C*R*N | 1 | 80.6 | 0.2467 |
| T*R*N | 1 | 80.6 | 0.5178 |
| C*T*R*N | 1 | 80.6 | 0.0943 |

^1^Treatment labels as in Table S1.
