# Supplementary material for: Responses of Grassland Production to Single and Multiple Global Environmental Changes
Source: PLoS Biol. 2005 Aug 9;3(10):e319. doi: 10.1371/journal.pbio.0030319 (PMC1182693; doi:10.1371/journal.pbio.0030319)
Supplement: Supplementary file 3 [file pbio.0030319.st003.doc]

Table S3. Results (*P*-values) from mixed model analyses of treatment effects on net primary production^1^

| Year | 1999 | 2000 | 2001 | 2002 | 2003 |
| --- | --- | --- | --- | --- | --- |
| Treatment |  |  |  |  |  |
| C | 0.7738 | 0.5152 | 0.1072 | 0.5069 | 0.2552 |
| T | 0.9313 | 0.7531 | 0.3248 | 0.7343 | 0.9441 |
| C*T | 0.4760 | 0.4139 | 0.9267 | 0.7434 | 0.8374 |
| R | 0.4332 | 0.4313 | 0.2735 | 0.5808 | 0.6462 |
| C*R | 0.7456 | 0.9797 | 0.4182 | 0.1758 | 0.9507 |
| T*R | 0.9699 | 0.3320 | 0.5038 | 0.7588 | 0.7667 |
| C*T*R | 0.8248 | 0.9724 | 0.3651 | 0.2175 | 0.7449 |
| N | **<0.0001** | 0.7054 | **<0.0001** | **0.0114** | **<0.0001** |
| C*N | 0.3989 | 0.9635 | *0.0675* | 0.7379 | 0.1765 |
| T*N | 0.6179 | *0.0832* | 0.7655 | 0.8700 | 0.3363 |
| C*T*N | 0.1588 | 0.8282 | 0.5392 | 0.1204 | 0.1417 |
| R*N | 0.5453 | 0.3244 | 0.8681 | 0.9422 | 0.2994 |
| C*R*N | 0.8518 | 0.4758 | *0.0656* | 0.8608 | 0.5855 |
| T*R*N | 0.3230 | 0.7321 | 0.6716 | 0.1400 | 0.2964 |
| C*T*R*N | 0.1680 | **0.0444** | 0.8900 | 0.4083 | 0.8202 |

^1^Treatment labels as in Table S1. Numerator degrees of freedom: 1 (all years). Denominator degrees of freedom (1999-2003): 23, 28, 28, 28, 27.
