# Supplementary material for: Responses of Grassland Production to Single and Multiple Global Environmental Changes
Source: PLoS Biol. 2005 Aug 9;3(10):e319. doi: 10.1371/journal.pbio.0030319 (PMC1182693; doi:10.1371/journal.pbio.0030319)
Supplement: Supplementary file 4 [file pbio.0030319.st004.doc]

Table S4. Results (*P*-values) from mixed model analyses of treatment effects on aboveground production^1^

| Year | 1999 | 2000 | 2001 | 2002 | 2003 |
| --- | --- | --- | --- | --- | --- |
| Treatment |  |  |  |  |  |
| C | 0.8849 | 0.3582 | 0.1883 | 0.8733 | 0.1507 |
| T | 0.9107 | 0.5273 | 0.2069 | 0.5313 | 0.8794 |
| C*T | 0.2359 | 0.9800 | 0.8788 | 0.7735 | 0.7308 |
| R | 0.2247 | 0.4581 | **0.0169** | 0.4661 | 0.6098 |
| C*R | 0.4631 | 0.7736 | 0.1559 | 0.9111 | 0.4725 |
| T*R | 0.8080 | 0.8786 | 0.2872 | 0.8987 | 0.8382 |
| C*T*R | 0.7951 | 0.9898 | 0.4704 | 0.3209 | 0.8685 |
| N | **0.0009** | 0.1588 | **0.0002** | **0.0004** | **<0.0001** |
| C*N | 0.4593 | 0.3712 | 0.1317 | 0.9734 | 0.2446 |
| T*N | 0.5376 | 0.5819 | 0.7625 | 0.6495 | 0.2460 |
| C*T*N | 0.3989 | 0.3660 | 0.2935 | 0.4766 | 0.1551 |
| R*N | 0.6472 | 0.4955 | 0.3686 | 0.8474 | 0.1432 |
| C*R*N | 0.9580 | 0.7530 | **0.0217** | 0.7513 | 0.8709 |
| T*R*N | 0.3675 | 0.3492 | 0.7994 | 0.2945 | 0.5322 |
| C*T*R*N | 0.1658 | *0.0721* | 0.5930 | 0.7507 | 0.9690 |

^1^Treatment labels as in Table S1. Numerator degrees of freedom: 1 (all years). Denominator degrees of freedom (1999-2003): 23, 28, 28, 28, 28.
