# Supplementary material for: Responses of Grassland Production to Single and Multiple Global Environmental Changes
Source: PLoS Biol. 2005 Aug 9;3(10):e319. doi: 10.1371/journal.pbio.0030319 (PMC1182693; doi:10.1371/journal.pbio.0030319)
Supplement: Supplementary file 5 [file pbio.0030319.st005.doc]

Table S5. Results (*P*-values) from mixed model analyses of treatment effects on belowground production^1^

| Year | 1999 | 2000 | 2001 | 2002 | 2003 |
| --- | --- | --- | --- | --- | --- |
| Treatment |  |  |  |  |  |
| C | 0.7449 | 0.9617 | 0.1960 | 0.1167 | 0.4813 |
| T | 0.6257 | 0.2204 | 0.7717 | 0.7027 | 0.6584 |
| C*T | 0.3404 | 0.2090 | 0.5570 | 0.7892 | 0.8148 |
| R | 0.2294 | *0.0753* | **0.0251** | 0.2292 | *0.0936* |
| C*R | 0.2327 | 0.7995 | 0.3456 | *0.0914* | 0.2810 |
| T*R | 0.3791 | 0.1436 | 0.5746 | 0.1073 | 0.7713 |
| C*T*R | 0.9385 | 0.9521 | 0.5255 | 0.6981 | 0.6859 |
| N | **0.0005** | 0.4216 | 0.1288 | 0.7120 | 0.7315 |
| C*N | 0.7776 | 0.4437 | 0.3366 | 0.9929 | 0.4607 |
| T*N | 0.7422 | *0.0623* | 0.9589 | 0.9825 | 0.9674 |
| C*T*N | *0.0658* | 0.5868 | 0.5178 | 0.1986 | 0.5997 |
| R*N | 0.5522 | 0.4694 | 0.1612 | 0.4936 | 0.4393 |
| C*R*N | 0.6477 | 0.2103 | 0.5593 | 0.9855 | 0.3598 |
| T*R*N | 0.6378 | 0.1870 | 0.1576 | 0.5068 | 0.1839 |
| C*T*R*N | 0.7841 | 0.2594 | 0.4520 | 0.2408 | 0.4554 |

^1^Treatment labels as in Table S1. Numerator degrees of freedom: 1 (all years). Denominator degrees of freedom (1999-2003): 23, 28, 28, 28, 27.
